# EnzymeDetector: an integrated enzyme function prediction tool and database

**DOI:** 10.1186/1471-2105-12-376

**Published:** 2011-09-23

**Authors:** Susanne Quester, Dietmar Schomburg

**Affiliations:** 1 Institute of Bioinformatics and Biochemistry, Technische Universität Braunschweig, Langer Kamp 19b, 38106 Braunschweig, Germany

## Abstract

**Background:**

The ability to accurately predict enzymatic functions is an essential prerequisite for the interpretation of cellular functions, and the reconstruction and analysis of metabolic models. Several biological databases exist that provide such information. However, in many cases these databases provide partly different and inconsistent genome annotations.

**Description:**

We analysed nine prokaryotic genomes and found about 70% inconsistencies in the enzyme predictions of the main annotation resources. Therefore, we implemented the annotation pipeline EnzymeDetector. This tool automatically compares and evaluates the assigned enzyme functions from the main annotation databases and supplements them with its own function prediction. This is based on a sequence similarity analysis, on manually created organism-specific enzyme information from BRENDA (Braunschweig Enzyme Database), and on sequence pattern searches.

**Conclusions:**

EnzymeDetector provides a fast and comprehensive overview of the available enzyme function annotations for a genome of interest. The web interface allows the user to work with customisable weighting schemes and cut-offs for the different prediction methods. These customised quality criteria can easily be applied, and the resulting annotation can be downloaded. The summarised view of all used annotation sources provides up-to-date information. Annotation errors that occur in only one of the databases can be recognised (because of their low relevance score). The results are stored in a database and can be accessed at http://enzymedetector.tu-bs.de.

## Background

A large number of online accessible biological databases provide genome annotations for a wide variety of organisms. Among the most frequently used resources are the RefSeq database from the National Center for Biotechnology Information (NCBI), the Kyoto Encyclopedia of Genes and Genomes (KEGG) [1-3], the PEDANT protein database [4-6], and the UniProtKB database [7]. In addition, specialised databases exist that focus on a specific group of organisms, for example the Pseudomonas Genome Database V2 [8] for Pseudomonas strains.

Hand-curated annotations are available only for well-investigated model organisms. To annotate the genomes of other organisms, the databases mainly use computational annotation tools with information on the implemented quality criteria being not always specified. There are obvious inconsistencies between pathway databases [9], and other databases providing predicted information on enzyme functions [10]. This is partly due to the fact that the automated annotation of enzyme functions is still a challenging task [11]. Additionally, the annotations may have been computed at different times, hence being based on different states of knowledge. In addition to the uncertainties introduced with gene prediction functional assignment often rely on dubious assignments arising from either errors made in manual annotations or transferred errors in automatic function predictions [10]. This leads to a high degree of inconsistency in the predicted enzyme functions.

In addition to the mentioned main annotation hosts, a number of annotation tools are available partly giving reliability scores, and some that integrate different sources. For example PRIAM [12] predicts enzyme functions based on sets of sequence profiles that have been computed for the entries of the ENZYME database, being an annotation source that may be integrated in a future version of EnzymeDetector. EFICAZ [13,14] is also based on residue patterns. It can be obtained as a stand-alone tool or accessed via a web interface. With EFICAZ it is possible to integrate annotation data from an external source. But only the data of the KEGG database can be integrated and no other sources.

Yang et al. [15] suggested an annotation confidence score based on sequence comparisons with some reference organisms. The tool presented by Chitale et al. [16]. delivers an annotation and a corresponding reliability score. As a serious drawback the user has to analyse the sequences one by one.

Within Apollo [17] and the UCSC Genome Browser database [18] it is possible to integrate annotation sources, but only with respect to the genomic positions of the genes and not on the available function predictions.

In order to easily access function annotations, life scientists currently have the choice between two different procedures. They either use one of the databases and may have to accept a serious loss of accuracy, or they manually compare different annotations. By selecting one data source, the result depends, among other factors, on the update cycle of the annotation host. Especially for the construction of metabolic models, the accuracy of the model strongly depends on the quality of the primary resources and the gene function prediction [19]. Even one missing enzyme function can be highly critical, because it might have a great impact on the whole model. As stated by Schnoes et. al., the annotation errors in public databases are a problem that should not be underestimated, since these errors are propagated over time [20]. In the manual evaluation of discrepancies between the sources, the scientist has no clear criteria for decision. In order to solve this problem and to give the scientist a fast overview, specialised tools that annotate, integrate, and mine the available information, are necessary [19].

For this purpose, the program EnzymeDetector was created. It includes a reasonable and comprehensible scoring scheme, and combines the information of the major databases, a frequently updated BLAST-based annotation, and a sequence pattern search. It provides the possibility to obtain a fast overview of the possible annotations for each gene and additional help to distinguish between their qualities. The advantage over previously described tools is given by the fact that the scientist does not have to manually analyse single sequences, but has the data for the whole genome pre-calculated in a database. Furthermore the database is easily accessible and can be downloaded. Although a background knowledge of functional annotation is very helpful, the tool EnzymeDetector can even be used by life scientists, not familiar with bioinformatics.

## Construction and content

**Figure 1:**
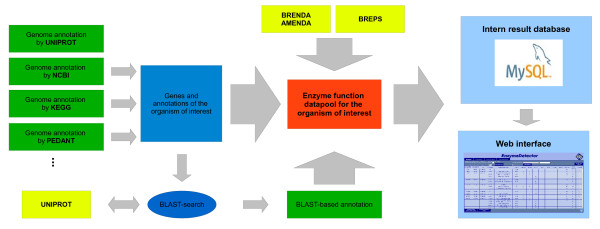
**Scheme of the work flow of EnzymeDetector**. The different annotations, shown in green, are either obtained from biological databases or obtained by a BLAST-based function prediction. As additional information sources BRENDA, AMENDA and BrEPS were included, shown in yellow. All informations together form the enzyme function data pool, shown in red. This data pool is stored in a SQL database, but can be accessed via a web interface as well.

An overview of the different parts of the EnzymeDetector program is shown in Figure 1.

We used nine different prokaryotic genomes as training data to determine optimal thresholds and default values. The statistics shown in this manuscript were done for those organisms as well. The nine organisms are *Corynebacterium glutamicum *ATCC13032, *Dinoroseobacter shibae *DFL12, *Escherichia coli *K12 MG1655, *Pseudomonas aeruginosa *PAO1, *Pseudomonas putida *KT2440, *Sulfolobus solfataricus *P2, *Thermus thermophilus *HB27, *Yersinia pseudotuberculosis *IP32953 and *Yersinia pseudotuberculosis *YPIII.

### Data collection

As a first step, the program collects and stores enzyme function annotations from different databases. Currently, the program uses data from NCBI, KEGG, PEDANT, a database specialised on *Sulfolobus *[21], the Pseudomonas Genome Database V2, and the annotation data found in Swiss-Prot [7]. The annotation of other databases can easily be added by including a respective parser.

### Additional annotation via a self-performed BLAST search against UniProtKB

As a second step, the program performs a BLAST analysis using all protein sequences of the organism as input sequences. The version 2.2.25 of the NCBI BLAST algorithm [22] is used. The search is performed against all protein sequences of the UniProt database [7]. The resulting hits are automatically evaluated, yielding the BLAST-based annotation.

Three criteria were taken into account for the evaluation of the BLAST hits:

- The **completeness of the Enzyme Commission numbers **(EC numbers): Incomplete EC numbers are ignored if other hits with complete EC numbers exist for the respective gene, because the necessary information on substrate specificity is not contained in incomplete EC numbers.

- The **expectation value **(E-value): For a conclusive function annotation the best BLAST has to have an E-value more than thirty orders of magnitude smaller than the E-value of the next best hit. If there are several hits presenting an E-value in the range of thirty orders of magnitude compared to the overall best hit, all of those hits are marked as candidates. Subsequently, these hits are assumed to be within the 'relevance range'. The value of thirty orders of magnitude was based on an evaluation of all BLAST hits of the nine organisms used as training data against the Swiss-Prot annotation. With the chosen value an optimal prediction was reached. About 99% of the enzymes annotated in Swiss-Prot were predicted in this way with only 7% of false positives (additional enzymatic activities for enzymes with a given EC-number in Swiss-Prot).

- The occurrence **of the EC numbers**: A cut-off value of 5 for the number of homologous sequences was chosen. If a certain EC number occurred more than 5 times in the list of all BLAST hits, it was considered to be relevant. This way, the inclusion of hits based on incorrectly annotated sequences is less likely. We chose a cut-off value of only 5 in order to prevent the loss of valuable information. With a manual analysis of the results of some BLAST searches, we found that with a higher cut-off value important information was lost. This information often proved to be crucial for model developers.

For every gene all EC numbers are stored, that are complete, within the 'relevance range' and have a relevant number of occurrence. If only hits with a low frequency were found, they were nevertheless accepted. This way new results were not rejected.

### Searching BRENDA and AMENDA

Specific experimental enzyme information from the enzyme databases BRENDA and from AMENDA [23] is added. The information in BRENDA is hand-curated and has a very high reliability. But the information is not connected to a specific enzyme sequence, if that information is not available in the original paper. This has to be considered analysing the EnzymeDetector result tables, which contain gene-enzyme combinations. When only a BRENDA/AMENDA annotation was found without a gene information, the result was marked as 'not sequence related'.

### Pattern search

The program BrEPS [24] performs a pattern-related enzyme annotation based on consensus sequence patterns. To analyse an organism, its protein sequences were searched against the pattern database, and the results were stored as additional information in the EnzymeDetector result database.

### Swiss-Prot

In the UniProtKB database of UniProt an ID mapping data file is stored. This file contains links between UniProt enzyme information and genes of different organisms. The information of the analysed organism was obtained and stored in the EnzymeDetector result database. Only information of the manually curated Swiss-Prot part of the database is used.

### Building the result database

The results of the procedure are stored in a relational database using MySQL, containing a combination of all collected and computed data. For each gene-enzyme combination found by the BLAST-search or present in one of the databases, an entry was created. For all entries three types of information are available:

- Gene-related information - gene identifier from NCBI (GI), the gene position, and the source organism

- Enzyme-related information - the EC number and the globally accepted name as defined by the IUBMB biochemical nomenclature committee

- Evaluation- related information - the E-value of the best BLAST hit of the enzyme, the position of the hit, the number of enzymes that are suggested for the gene by the BLAST evaluation program, information on the number of databases that predicted the particular enzyme, and whether the enzyme is confirmed by the pattern-search program BrEPS.

A default scoring scheme was constructed for the weights of the different data sources based on a comparison with the manual Swiss-Prot annotation for the respective gene (as far as this was available). Precision (= 100 * true positives/(true positives + false positives)) and recall (= 100 * true positives/(true positives + false negatives)) of the sources were calculated. The default values for the sources were calculated based on the average F1-scores (= 2 * (precision * recall)/(precision + recall)). We set the relevance scores of the different sources in relation to the relevance score of the BLAST-based annotation. For a F1-score of 100% a relevance score of 13 is assigned, for a F1 score between 95 and 100% a score of 12, and for any other value the relevance score drops by one for each drop of the F1-score by 5%, leading to values of zero for F1-scores < 40%. These values were chosen relative to the top score of 8 for the BLAST-based annotation. This is a constraint arising from the classification of the BLAST results in 8 different groups. The other scores were defined dependent on that.

**Figure 2:**
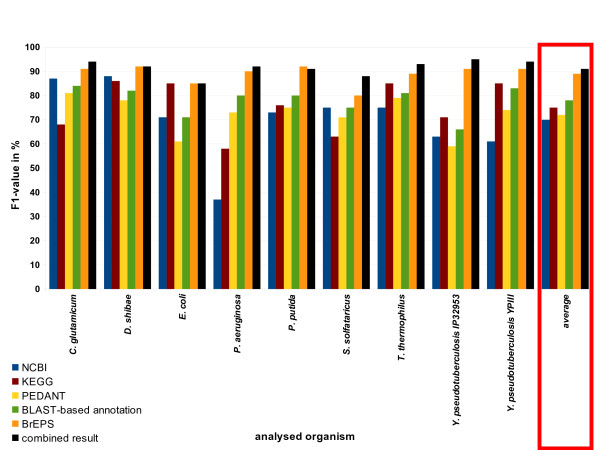
**F1-score of the different annotation sources**. The F1-score of the different data sources is shown in different colours (NCBI - blue, KEGG - yellow, PEDANT - red, BLAST-based annotation - green, BrEPS - orange). The F1-score of the combination of all sources is shown in black. The evaluation was performed against Swiss-Prot.

In Figure 2 the F1-scores of the different sources are shown. Additionally, the score of the combined information is shown in black, which is considerably higher than the score of any single source. Only the pattern-based BrEPS annotation has higher values for some of the organisms, but gives predictions only for 12% of the gene products annotated as enzymes. The fact that in some cases the combined result of the EnzymeDetector shows a lower agreement with the Swiss-Prot annotations than BrEPS, is based on the fact that the BrEPS can be overruled by the combined result of several other annotation sources.

According to the grouping of the F1-scores and the average F1 of the different databases, KEGG and PEDANT were assigned a default value of 7, and PEDANT and NCBI default values of 6.

For the BLAST-based method according to the average F1 value a top score of 8 was determined. This score consists of two parts - on the one hand the score for the best E-value of the annotation found in general, i.e. in the whole UniProt database with TrEMBL included, and on the other hand the score of the best E-value found in the reviewed Swiss-Prot part. The overall score for the BLAST-based annotation is built by the sum of these two scores. The individual score is achieved by the classification of the quality measures in four groups: Annotations with an E-value greater than 10^-40 ^were assigned a score of 1. Those with E-values in the range from 10^-40 ^to 10^-80 ^were assigned a score of 2. For E-values ranging from 10^-80 ^to 10^-120 ^a score of 3 was added and for E-values smaller than 10^-120 ^a score of 4.

For the BrEPS evaluation a top score between 1 and 10 was assigned depending on the quality measure calculated from the program BrEPS.

For hand-curated data (e.g. Swiss-Prot and BRENDA) we assigned a score of 50. This value was chosen because it is considerably higher than the sum of the values of all other sources. This means that the hand-curated data cannot be overruled by other sources in the comparison process.

A score of 25 was assigned to AMENDA. Although the information in AMENDA has a high reliability, it is based on a text-mining process. Thus, the data is not as certain as hand-curated data.

Swiss-Prot was chosen as standard of truth, because it has a large number of manually curated function assignments over a wide range of organisms. In all probability the different sources synchronise their annotation data with those in Swiss-Prot in constant intervals. Thereby, the F1-score of the annotation predictions for those genes where no Swiss-Prot entry is available is most certainly not as high as for the genes we analysed. Lacking an alternative for the determination of the ranking of the sources, we had to rely on the F1-scores determined against Swiss-Prot. It should be noted, that because the BLAST-based annotation is performed against UniProt and the query sequence is not excluded from the search results, the Swiss-Prot annotations get included in the evaluated results. But this is balanced by the fact that we not only use the E-values as a decision criterion, but the number of occurrences of an EC-number among the BLAST-hits as well. Thereby, even if the query sequence is found with a very good E-value, it will only be considered as a candidate if other sequences with that annotation match the search sequence as well.

The sum of all different relevance values define the overall-relevance of a result entry - the overall relevance score marking the quality of the annotation.

### Evaluation of function predictions

The following statistics were done for the nine organisms mentioned above. For the analysed organisms on average an enzyme function was predicted for 30% of its genes (Table 1), using annotations that had an overall relevance score of at least 7.

**Table 1:** Percentages of genes with predicted enzyme functions.

Organism	Percentage of genes with predicted enzyme function
*C. glutamicum*	29%

*D. shibae*	36%

*E. coli*	47%

*P. aeruginosa*	27%

*P. putida*	26%

*S. solfataricus*	25%

*T. thermophilus*	27%

*Y. pseudotuberculosis *IP32953	26%

*Y. pseudotuberculosis *YPIII	31%

*average*	30%

This enzyme content matches the generally accepted value. As a reference value we took the *Escherichia coli *enzyme content of 35% as given by Swiss-Prot. We took *E. coli *as reference because it is one of the best-analysed organisms.

**Figure 3:**
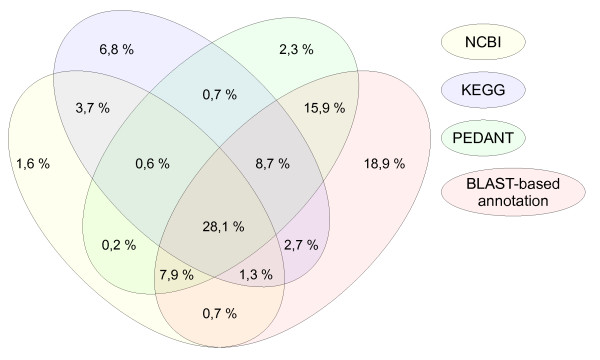
**Venn diagram indicating the agreement between annotations in the different databases**. The annotations found in NCBI are represented by the yellow ellipse, KEGG annotations by the blue one, PEDANT annotations by the green one, and those found in the BLAST-based annotation in red. For the BLAST-based annotation only hits were considered that had a maximal E-value of 10^-80^.

In only 29% of all annotations, the three main annotation databases predicted identical enzyme functions. For another 14% there was agreement between two of the three sources, and for 30% of all annotated genes only one of the three databases contained a function assignment at all (Figure 3). On average 19% of all genes with a predicted enzyme function were only annotated by the BLAST-based annotation and not in any of the main annotation databases. For the BLAST-based annotation, only hits with an E-value lower than 10^-80 ^were considered. The additional BLAST results can be explained by the fact, that the annotation of the other annotation sources may be based on earlier UniProt versions, or that different assignment criteria were used. The different annotation sites provide no information on the time period between updates of their annotations.

**Figure 4:**
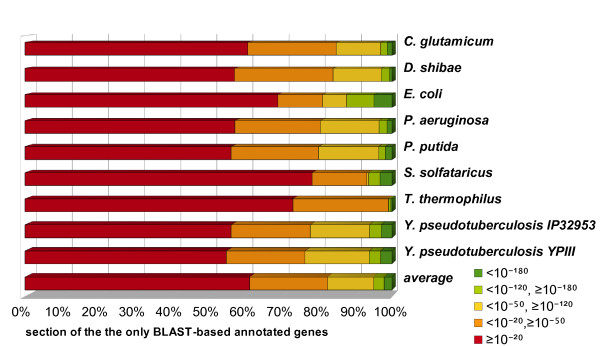
**E-value categories of the result database entries that were only found by the BLAST-based annotation**. The database entries are grouped by their E-value: The best hits are shown in dark green with E-values smaller than 10^-180, ^good hits are shown in light green with Evalues between 10^-180 ^and 10^-120^, possibly significant hits with E-values between 10^-50 ^and 10^-120 ^are shown in orange, and dubious hits with E-values larger than 10^-20 ^are shown in red.

On average 13% of all additional annotations, added by the BLAST-based annotation, had a low E-value between 10^-50 ^and 10^-120 ^(Figure 4). 5% even had a very low E-value of <10^-120^. The 21% of annotations with E-values between 10^-20 ^and 10^-50 ^represent promising candidates if an enzyme function is missing for the construction of a metabolic model. 61% of the annotations have an E-value higher than 10^-20^. These hits get a low relevance score and are thereby excluded, if an adequate cut-off is chosen. As expected, the function predictions for the hyperthermophilic archaeon *Sulfolobus solfataricus *had a lower average quality than for the analysed bacteria, reflecting the small number of reliable enzyme sequences of Archaea and the highly specialised metabolism. Therefore, the BLAST hits displayed much higher average E-values.

**Figure 5:**
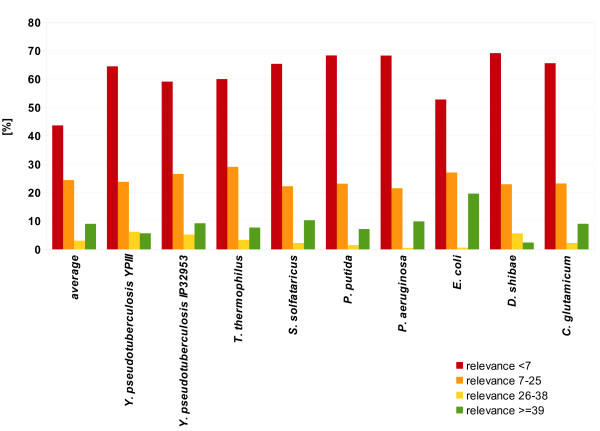
**EnzymeDetector relevance groups**. The overall relevances of all evaluated organisms and the mean are shown merged into four groups according to the different cut-off suggestions we make. Only the best candidate for every gene was considered. Genes beneath the minimal cut-off of 7 are shown in red (overall-relevance 1-6). Genes with a good confidence level are shown in orange (overall-relevance 7-25), genes with a very good confidence level in yellow (overall-relevance 26-38). Annotations of the top scoring group with the highest confidence are shown in green (overall-relevance >38).

We grouped the overall relevance of the EnzymeDetector results in four categories (Figure 5). We created these groups according to the three different cut-offs we suggest further down. For every gene only the best candidate was considered for this evaluation.

1. Annotations with an overall-relevance smaller than 7 (i.e. beneath the minimal cut-off we suggest) are shown in red. An average of 64% of all genes belong to that group, resulting mainly from BLAST hits with an intermediate E-value.

2. Qualitatively good annotations with an overall-relevance between 7 and 25 are shown in orange. 24% of the results can be found in this group. If an annotations has an overall-score in the lower range of this group, it was only found in one of the annotation sources and therefore might have to be checked by the scientist.

3. Annotations with a very good confidence are shown in yellow. Their overall-relevance is between 26 and 38. Those hits have a perfect recall and a precision of over 95%. 3% of the results belong to this group.

4. Annotations in the top-scoring group have an overall-relevance greater than 38. This group is shown in green. On average, 6% of the results belong to that group.

As expected the results for *E. coli *have the highest relevance scores. This is due to the fact that it is an experimentally very well-analysed organism with reliable annotations in the input databases, which yields high overall relevance.

**Figure 6:**
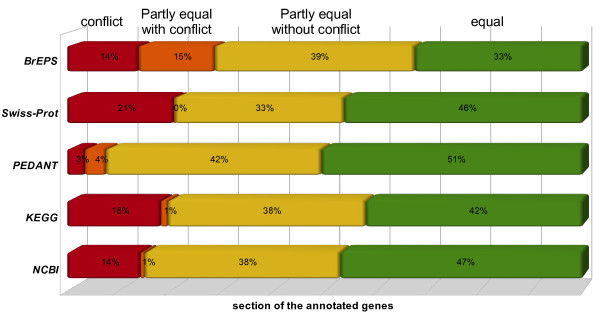
**Agreement of annotations from the BLAST-based annotation with the annotations from the input databases**. Only the genes were taken into account that had an annotation in the BLAST-based annotation set and in the according input database. Annotations that are completely equal are presented in green. Annotations that were partly equal without a conflict are shown in yellow. Partly equal annotations with a conflict are shown in orange and conflicting annotations are shown in red.

If a gene annotation was found by the BLAST-based annotation and in at least one of the other sources, the prediction was identical in most of the cases (Figure 6). As an example, the PEDANT and the BLAST-based annotations were identical in 51% of all cases, and in another 42% of the annotations, non-conflicting evidence was obtained (for example, the gene b0004 of *E. coli K12 *had an enzyme function of 4.2.3.1 in the BLAST-based annotation, while it was annotated as 4.2.3.1 and 4.2.99.2 in PEDANT). In only 7% of all cases, the annotations either disagreed in part (for example, gene b2799 of *E.coli: *1.1.1.77, 1.1.1.202, and 1.1.1.1 annotated in PEDANT and 1.1.1.77, 1.1.1.202, and 2.7.13.3 annotated in the BLAST-based annotation), or there was a full disagreement (for example, gene b2717 of *E.coli*: 3.4.23.51 annotated in the BLAST-based annotation and 1.12.98.1 annotated in PEDANT).

## Utility

The EnzymeDetector website holds a database containing the described combined enzyme annotations. This database will be updated twice a year to keep the data up-to-date. The results are presented via a web interface, which allows the user to interactively explore, process, and download the data. In the current version, all prokaryotic organisms are included in the database, with the genome annotations from NCBI, KEGG, PEDANT, and Swiss-Prot, and the BRENDA and AMENDA data included. The BLAST-based annotation is added continuously (limited by available computer time). This may lead to the fact that no E-value information is provided for some organisms, and that for those organisms the highest reachable overall-relevance is smaller compared to those with a BLAST-based annotation.

An interactive help is displayed by selection of the help sign in the lower right corner of every site. Subsequently, a help or explanation window opens when the cursor is pointed at any object.

The organism can be selected by the user on the start page of the web interface. After this selection, the annotation sources currently available for that organism are displayed. Annotation sources to be included in the analysis can be selected. The default relevance scores for those sources are given and can be modified.

**Figure 7:**
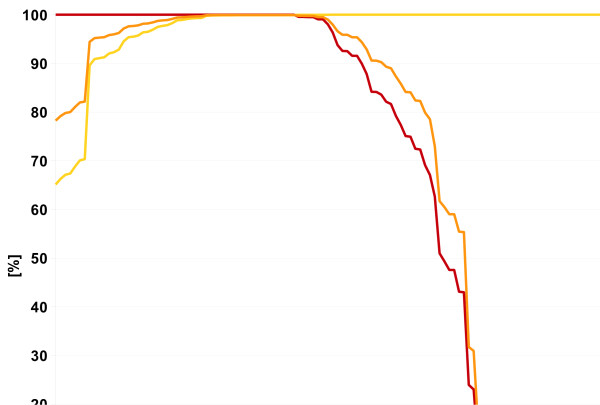
**Average precision, recall and F1-score for the different overall relevances**. The average precision is shown in yellow, the average recall is shown in red, and the F1-score is shown in orange. The values were calculated for 81 different organisms excluding the nine organisms of the training set.

Additionally, the user can select default cut-off values for the extraction of the data from the result pool. We suggest tree different cut-offs depending on the quality of data the user wants to achieve. The recommended cut-off scheme is based on Figure 7. The cut-offs were defined by evaluation of the results of 81 analysed organisms (excluding the nine organisms representing the training data) against the accordant Swiss-Prot annotations (list of organisms can be found in additional file 1).

- For generous filtering we suggest a cut-off of 7. With this value the retrieved data has optimal recall, but a low precision. With this setting genes that are only annotated by the BLAST-based annotation (with a quality score of 7 and higher) are not lost.

- For medium filtering we suggest a cut-off of 26. This is the lowest relevance score for which the average F1 is greater than 99%.

If maximum precision is wanted we suggest a cut-off of 39. This is the lowest relevance for which the F1 is maximal.

By default the cut-off value for the overall-relevance is set to the generous filtering option on the web interface. This can be changed by the user at any time.

The cut-off for the maximal E-value is set to 10^-25^. This cut-off only affects the data of the BLAST-based annotation. Only results with an E-value below the chosen cut-off are integrated in the BLAST-based annotation.

Both cut-off values can be changed at any time of the analysis.

On the web interface the user has the choice between four different views on the data:

### The tabular view (Figure 8)

**Figure 8:**
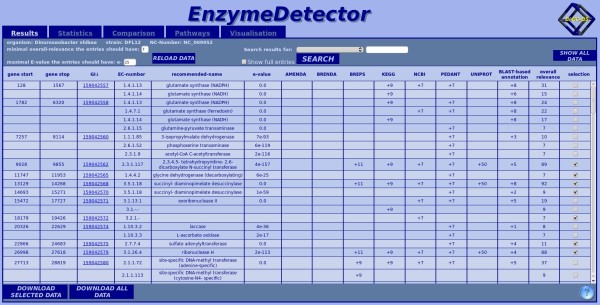
**Tabular view of the web interface**. In this case the results for *Dinoroseobacter shibae *strain DFL12 are presented. In the table a summary of our own result database is shown. For every gene-enzyme combination, a new data row is created with information about the gene (positions, GI), with information to the found annotation (recommended name, EC number, best E-value of the found annotation) and with information on the quality of the annotation (relevances of the input databases, overall relevance).

By default, all columns are sorted by gene identifier. The user can sort the entries by EC number or accepted name by clicking on the respective column headers. It is possible to search the result table for a certain entry by using the search mask. The possible search fields are GI, gene position, EC number, and recommended name. Additionally, it is possible to filter the results for data source occurrence.

The cut-off values that are used for filtering the displayed data can be adapted at any time. If just one candidate for a gene within the selected constraints is available, the entry is automatically selected. If there are conflicting EC annotations, the user has to decide which annotation/s to select.

The selected subset of data or the whole dataset can be downloaded as a CSV file for further processing.

### The statistics view (Figure 9)

**Figure 9:**
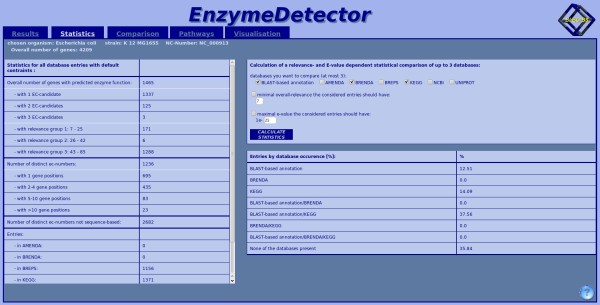
**Statistics view of the web interface**. Here shown for *Escherichia coli*. In the left part of the site, the static view is shown, calculated for the default cut-off values and in the right part of the site users have the possibility to assign their own cut-offs and calculate the statistics with these.

By clicking on the corresponding tab, the user can switch to the statistics. The page is split into two parts - the static and the dynamic view. For the static view the whole dataset with default constraints is used. The dynamic view presents basically the same information, but the computation considers only those data entries that fulfil the user-chosen constraints. The selectable constraints are the minimal overall-relevance and the maximal E-value. Additionally, the user has the possibility to compare up to three of the annotation sources to obtain their degree of consistency.

### The annotation comparison view (Figure 10)

**Figure 10:**
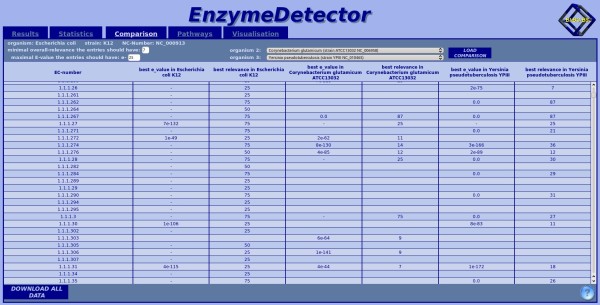
**Display of the annotation comparison view of the web interface**. Here calculated for *Escherichia coli*, *Corynebacterim glutamicum *and *Yersinia pseudotuberculosis*. The enzyme stock of the calculated organism is shown in comparison to up to two other organisms. For each organism the best overall-relevance and the best E-value is shown.

In this view, the user has the possibility to compare the enzyme stock of the explored organism to that of one or two other organisms. All enzymes of the explored organisms are displayed together with their best E-value and their best overall relevance. All data sets can be downloaded.

### The Pathway view (Figure 11)

**Figure 11:**
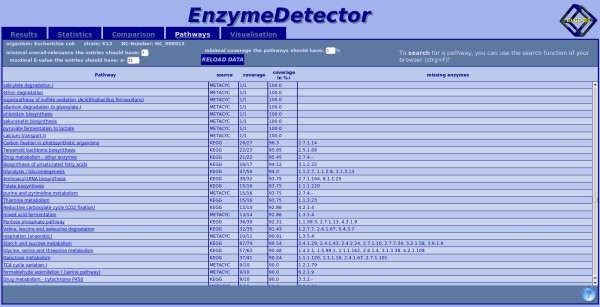
**Display of the pathway view of the web interface**. Here shown for *Escherichia coli*. The pathway names are shown together with their source (KEGG or Metacyc), their coverage and the enzymes that are missing.

The pathway view shows a list of all pathways from MetaCyc [25] and KEGG. The total number of enzymes in the pathway and the number of found enzymes are displayed. The enzymes that are missing are given as well. By default the table is sorted by pathway name, but it can also be sorted by the source or the coverage.

### Outlook

A user upload field is planned. Thus, the user can upload an own annotation of the provided organism (in a defined format). This information will be integrated in the result of the web interface.

## Discussion

The evaluation of the EnzymeDetector results clearly shows that reliance on only one annotation source cause in loss of valuable information. In only one third the big annotations host agree in their annotation. 19% of the annotations found by the EnzymeDetector were even just found by the performed BLAST-search.

The results of the EnzymeDetector help the user to find all information available for a genome and helps him to distinguish between the qualities of the annotations. The provided data of the web interface will be used by life scientists for obtaining information on a selected organism or gene of interest. Furthermore, the tool is certainly helpful for developers of metabolic models, providing more reliable information on the enzymes present in defined organisms.

## Conclusions

For the detailed analysis of the metabolism of an organism, it is essential to have an accurate annotation of enzyme functions. Given that there are inconsistencies and errors in the existing databases, it is not recommended to rely on only one source. Hence, it is beneficial to integrate and compare the existing genome annotations of different sources. However, it is extremely time-consuming, if not impossible, to manually integrate all existing function predictions. Therefore, we provide the tool EnzymeDetector, which gives a fast and up-to-date overview of the available annotation data from a selected set of sources. In addition, it ranks the information by quality. The results are accessible via a web interface. Thus, it is easy for model developers or lab scientists to gain information about a gene of interest or the whole enzyme stock of an organism. It is possible to assign a personal scoring scheme to the different annotation sources. This way a customised data set can be created. All information is downloadable in CSV format. Hence, the user can easily perform a detailed analysis with the data. An option will be added that allows the user to upload data from other sources in a predefined format. This will facilitate the integration of organism-specific databases, which improves the overall results.

Because the program performs a BLAST-search, the EnzymeDetector approach clearly shows better results for well-curated genomes like *Escherichia coli*. Clearly function assignment to genes based on that search is more significant with genes that have similarities to many known sequences.

The thresholds suggested in this paper are based on the analysis of nine organisms. These values will be regularly updated with analysis of the information of more organisms. Thus the threshold values will get more accurate or rather more adaptive to all organisms over time.

Currently EnzymeDetector results are only available for prokaryotes. The integration of eukaryotes is planned in the future.

## Availability and requirements

Project name: EnzymeDetector web interface;

Project home page: http://enzymedetector.tu-bs.de or http://edbs.tu-bs.de;

Operating system: Platform independent

Programming language: python, JavaScript, html

## Authors' contributions

SQ developed the software, carried out the validations, created the web interface and drafted the manuscript. DS had the original idea and supervised the work. All authors read and approved the final manuscript.

## Supplementary Material

Additional file 1 **List of evaluated organisms**. A list of all organisms that were evaluated by the EnzymeDetector including a BLAST-based annotation. Click here for file

## References

[B1] Kanehisa M Goto S Hattori M Aoki-Kinoshita KF Itoh M Kawashima S Katayama T Araki M Hirakawa M From genomics to chemical genomics: new developments in KEGG Nucleic Acids Res 2006 34 D354 357 10.1093/nar/gkj102 16381885 PMC1347464

[B2] Kanehisa M Goto S Furumichi M Tanabe M Hirakawa M KEGG for representation and analysis of molecular networks involving diseases and drugs Nucleic Acids Res 2010 38 D355 360 10.1093/nar/gkp896 19880382 PMC2808910

[B3] Kanehisa M Goto S KEGG: kyoto encyclopedia of genes and genomes Nucleic Acids Res 2000 28 27 30 10.1093/nar/28.1.27 10592173 PMC102409

[B4] Walter MC Rattei T Arnold R Güldener U Münsterkötter M Nenova K Kastenmüller G Tischler P Wölling A Volz A Pongratz N Jost R Mewes H-W Frishman D PEDANT covers all complete RefSeq genomes Nucleic Acids Res 2009 37 D408 411 10.1093/nar/gkn749 18940859 PMC2686588

[B5] Riley ML Schmidt T Artamonova II Wagner C Volz A Heumann K Mewes H-W Frishman D PEDANT genome database: 10 years online Nucleic Acids Res 2007 35 D354 357 10.1093/nar/gkl1005 17148486 PMC1761421

[B6] Frishman D Mokrejs M Kosykh D Kastenmüller G Kolesov G Zubrzycki I Gruber C Geier B Kaps A Albermann K Volz A Wagner C Fellenberg M Heumann K Mewes H-W The PEDANT genome database Nucleic Acids Res 2003 31 207 211 10.1093/nar/gkg005 12519983 PMC165452

[B7] Ongoing and future developments at the Universal Protein Resource Nucleic Acids Res 2011 39 D214 219 21051339 10.1093/nar/gkq1020 PMC3013648

[B8] Winsor GL Van Rossum T Lo R Khaira B Whiteside MD Hancock REW Brinkman FSL Pseudomonas Genome Database: facilitating user-friendly, comprehensive comparisons of microbial genomes Nucleic Acids Res 2009 37 D483 488 10.1093/nar/gkn861 18978025 PMC2686508

[B9] Soh D Dong D Guo Y Wong L Consistency, comprehensiveness, and compatibility of pathway databases BMC Bioinformatics 2010 11 449 10.1186/1471-2105-11-449 20819233 PMC2944280

[B10] Poptsova MS Gogarten JP Using comparative genome analysis to identify problems in annotated microbial genomes Microbiology (Reading, Engl.) 2010 156 1909 1917 10.1099/mic.0.033811-0 20430813

[B11] Furnham N Garavelli JS Apweiler R Thornton JM Missing in action: enzyme functional annotations in biological databases Nat Chem Biol 2009 5 521 525 19620987 10.1038/nchembio0809-521

[B12] Claudel-Renard C Chevalet C Faraut T Kahn D Enzyme-specific profiles for genome annotation: PRIAM Nucleic Acids Res 2003 31 6633 6639 10.1093/nar/gkg847 14602924 PMC275543

[B13] Tian W Arakaki AK Skolnick J EFICAz: a comprehensive approach for accurate genome-scale enzyme function inference Nucleic Acids Res 2004 32 6226 6239 10.1093/nar/gkh956 15576349 PMC535665

[B14] Arakaki AK Huang Y Skolnick J EFICAz2: enzyme function inference by a combined approach enhanced by machine learning BMC Bioinformatics 2009 10 107 10.1186/1471-2105-10-107 19361344 PMC2670841

[B15] Yang Y Gilbert D Kim S Annotation confidence score for genome annotation: a genome comparison approach Bioinformatics 2010 26 22 29 10.1093/bioinformatics/btp613 19855104

[B16] Chitale M Hawkins T Park C Kihara D ESG: extended similarity group method for automated protein function prediction Bioinformatics 2009 25 1739 1745 10.1093/bioinformatics/btp309 19435743 PMC2705228

[B17] Misra S Harris N Using Apollo to browse and edit genome annotations Curr Protoc Bioinformatics 2006 Chapter 9 Unit 9.5 18428771 10.1002/0471250953.bi0905s12

[B18] Fujita PA Rhead B Zweig AS Hinrichs AS Karolchik D Cline MS Goldman M Barber GP Clawson H Coelho A Diekhans M Dreszer TR Giardine BM Harte RA Hillman-Jackson J Hsu F Kirkup V Kuhn RM Learned K Li CH Meyer LR Pohl A Raney BJ Rosenbloom KR Smith KE Haussler D Kent WJ The UCSC Genome Browser database: update 2011 Nucleic Acids Res 2010 10.1093/nar/gkq963 PMC3242726 20959295

[B19] Médigue C Moszer I Annotation, comparison and databases for hundreds of bacterial genomes Res Microbiol 2007 158 724 736 10.1016/j.resmic.2007.09.009 18031997

[B20] Schnoes AM Brown SD Dodevski I Babbitt PC Annotation error in public databases: misannotation of molecular function in enzyme superfamilies PLoS Comput Biol 2009 5 e1000605 10.1371/journal.pcbi.1000605 20011109 PMC2781113

[B21] She Q Singh RK Confalonieri F Zivanovic Y Allard G Awayez MJ Chan-Weiher CC Clausen IG Curtis BA De Moors A Erauso G Fletcher C Gordon PM Heikamp-de Jong I Jeffries AC Kozera CJ Medina N Peng X Thi-Ngoc HP Redder P Schenk ME Theriault C Tolstrup N Charlebois RL Doolittle WF Duguet M Gaasterland T Garrett RA Ragan MA Sensen CW Van der Oost J The complete genome of the crenarchaeon Sulfolobus solfataricus P2 Proc Natl Acad Sci USA 2001 98 7835 7840 10.1073/pnas.141222098 11427726 PMC35428

[B22] Camacho C Coulouris G Avagyan V Ma N Papadopoulos J Bealer K Madden TL BLAST+: architecture and applications BMC Bioinformatics 2009 10 421 10.1186/1471-2105-10-421 20003500 PMC2803857

[B23] Chang A Scheer M Grote A Schomburg I Schomburg D BRENDA, AMENDA and FRENDA the enzyme information system: new content and tools in 2009 Nucleic Acids Res 2009 37 D588 592 10.1093/nar/gkn820 18984617 PMC2686525

[B24] Bannert C Welfle A Aus dem Spring C Schomburg D BrEPS: A flexible and automatic protocol to compute enzyme-specific sequence profiles for functional annotation BMC Bioinformatics 2010 11 589 10.1186/1471-2105-11-589 21122127 PMC3009691

[B25] Caspi R Altman T Dale JM Dreher K Fulcher CA Gilham F Kaipa P Karthikeyan AS Kothari A Krummenacker M Latendresse M Mueller LA Paley S Popescu L Pujar A Shearer AG Zhang P Karp PD The MetaCyc database of metabolic pathways and enzymes and the BioCyc collection of pathway/genome databases Nucleic Acids Res 2010 38 D473 479 10.1093/nar/gkp875 19850718 PMC2808959

