# Supplementary material for: EnzymeDetector: an integrated enzyme function prediction tool and database
Source: BMC Bioinformatics. 2011 Sep 23;12:376. doi: 10.1186/1471-2105-12-376 (PMC3224133; doi:10.1186/1471-2105-12-376)
Supplement: Additional file 1 — List of evaluated organisms. A list of all organisms that were evaluated by the EnzymeDetector including a BLAST-based annotation. [file 1471-2105-12-376-S1.PDF]

evaluated organisms

genus	strain
Dinoroseobacter_shibae	DFL_12
Pseudomonas_aeruginosa	PA01
Pseudomonas_putida	KT2440
Sulfolobus_solfataricus	P2
Thermus_thermophilus	HB27
Yersinia_pseudotuberculosis	YPIII
Corynebacterium_glutamicum	ATCC_13032
Yersinia_pseudotuberculosis	IP32953
Escherichia_coli	K_12_MG1655
Escherichia_coli	O157:H7_Sakai
Sulfolobus_tokodaii	strain7
Yersinia_pestis	CO92
Escherichia_coli	K_12_W3110
Corynebacterium_glutamicum	ATCC_13032
Escherichia_coli	O6:K2:H1_CFT073
Pseudomonas_syringae	pv_tomato_DC3000
Corynebacterium_diphtheriae	gravis_NCTC13129
Rhodopseudomonas_palustris	CGA009
Yersinia_pestis	91001
Thermus_thermophilus	HB8
Pseudomonas_syringae	pv_syringae_B728a
Pseudomonas_fluorescens	Pf_5
Sulfolobus_acidocaldarius	DSM_639
Corynebacterium_jeikeium	K411
Pseudomonas_syringae	pv_phaseolicola_1448A
Rhodopseudomonas_palustris	HaA2
Rhodopseudomonas_palustris	BisB18
Escherichia_coli	O18:K1:H7_UTI89
Rhodopseudomonas_palustris	BisB5
Pseudomonas_entomophila	L48
Yersinia_pestis	Antiqua
Yersinia_pestis	Nepal516
Escherichia_coli	O6:K15:H31_536
Rhodopseudomonas_palustris	BisA53
Pseudomonas_aeruginosa	UCBPP_PA14
Escherichia_coli	O1:K1:H7
Hyperthermus_butylicus	DSM_5456
Yersinia_enterocolitica	subsp_enterocolitica_8081
Staphylothermus_marinus	F1
Corynebacterium_glutamicum	R
Yersinia_pestis	Pestoides_F
Pseudomonas_mendocina	ymp
Pseudomonas_stutzeri	A1501
Pseudomonas_putida	F1
Pseudomonas_aeruginosa	PA7
Yersinia_pseudotuberculosis	IP31758
Escherichia_coli	O139:H28_E24377A
Escherichia_coli	O9_HS
Yersinia_pestis	Angola
Pseudomonas_putida	GB_1
Escherichia_coli	K_12_DH10B
Escherichia_coli	SMS_3_5
Pseudomonas_putida	W619
Corynebacterium_urealyticum	DSM_7109
Escherichia_coli	C_ATCC_8739
Rhodopseudomonas_palustris	TIE_1
Escherichia_coli	O157:H7_EC4115
Escherichia_coli	O152:H28_SE11
Escherichia_fergusonii	ATCC_35469
Pseudomonas_aeruginosa	LESB58
Escherichia_coli	55989
Escherichia_coli	O81_D1a
Escherichia_coli	O8_IAI1
Escherichia_coli	O7:K1_IAI39

evaluated organisms

genus	strain
Escherichia_coli	O45:K1:H7_S88
Escherichia_coli	O17:K52:H18_UMN026
Corynebacterium_aurimucosum	ATCC_700975
Sulfolobus_islandicus	LS215
Sulfolobus_islandicus	M1425
Pseudomonas_fluorescens	SBW25
Sulfolobus_islandicus	M1627
Sulfolobus_islandicus	YG5714
Sulfolobus_islandicus	YN1551
Sulfolobus_islandicus	M164
Escherichia_coli	BW2952
Escherichia_coli	BL21_Gold
Escherichia_coli	B_REL606
Escherichia_coli	O157:H7_TW14359
Escherichia_coli	O103:H2_12009
Escherichia_coli	O111:H__11128
Escherichia_coli	O26:H11_11368
Rhodothermus_marinus	DSM_4252
Sulfolobus_islandicus	LD85
Escherichia_coli	O55:H7_CB9615
Meiothermus_ruber	DSM_1279
Bacillus_megaterium	QM_B1551
Yersinia_pestis	Z176003
Bacillus_megaterium	DSM_319
Staphylothermus_hellenicus	DSM_12710
Syntrophothermus_lipocalidus	DSM_12680
